# Impaired Glucose Metabolism among Those with and without Diagnosed Diabetes and Mortality: A Cohort Study Using Health Survey for England Data

**DOI:** 10.1371/journal.pone.0119882

**Published:** 2015-03-18

**Authors:** Vanessa L. Z. Gordon-Dseagu, Jennifer S. Mindell, Andrew Steptoe, Alison Moody, Jane Wardle, Panayotes Demakakos, Nicola J. Shelton

**Affiliations:** Department of Epidemiology and Public Health, UCL (University College London), 1–19 Torrington Place, London, WC1E 6BT, United Kingdom; Indiana University School of Medicine, UNITED STATES

## Abstract

**Background:**

The extent that controlled diabetes impacts upon mortality, compared with uncontrolled diabetes, and how pre-diabetes alters mortality risk remain issues requiring clarification.

**Methods:**

We carried out a cohort study of 22,106 Health Survey for England participants with a HbA1_C_ measurement linked with UK mortality records. We estimated hazard ratios (HRs) of all-cause, cancer and cardiovascular disease (CVD) mortality and 95% confidence intervals (CI) using Cox regression.

**Results:**

Average follow-up time was seven years and there were 1,509 deaths within the sample. Compared with the non-diabetic and normoglycaemic group (HbA1_C_ <5.7% [<39mmol/mol] and did not indicate diabetes), undiagnosed diabetes (HbA1_C_ ≥6.5% [≥48mmol/mol] and did not indicate diabetes) inferred an increased risk of mortality for all-causes (HR 1.40, 1.09–1.80) and CVD (1.99, 1.35–2.94), as did uncontrolled diabetes (diagnosed diabetes and HbA1_C_ ≥6.5% [≥48mmol/mol]) and diabetes with moderately raised HbA1_C_ (diagnosed diabetes and HbA1_C_ 5.7-<6.5% [39-<48mmol/mol]). Those with controlled diabetes (diagnosed diabetes and HbA<5.7% [<39mmol/mol]) had an increased HR in relation to mortality from CVD only. Pre-diabetes (those who did not indicate diagnosed diabetes and HbA1_C_ 5.7-<6.5% [39-<48mmol/mol]) was not associated with increased mortality, and raised HbA1_C_ did not appear to have a statistically significant impact upon cancer mortality. Adjustment for BMI and socioeconomic status had a limited impact upon our results. We also found women had a higher all-cause and CVD mortality risk compared with men.

**Conclusions:**

We found higher rates of all-cause and CVD mortality among those with raised HbA1_C_, but not for those with pre-diabetes, compared with those without diabetes. This excess differed by sex and diabetes status. The large number of deaths from cancer and CVD globally suggests that controlling blood glucose levels and policies to prevent hyperglycaemia should be considered public health priorities.

## Introduction

The World Health Organization estimates that annually 3.4 million deaths are due to high levels of glucose in the blood.[1] The prevalence of diabetes, the disease most closely associated with chronic hyperglycaemia, currently totals 366 million worldwide and this figure is expected to rise to over 550 million by 2030.[2] In England, around 3.1 million adults over the age of 16 are estimated to be living with diabetes, equating to 7.4% of the population, and the Association of Public Health Observatories projected that this will increase to 4.6 million (9.5%) by 2030.[3] Glycated haemoglobin (HbA1_C_) is a reliable biomarker of long term blood glucose levels. The International Expert Committee recommended in 2009 that HbA1_C_ is appropriate for the clinical diagnosis of diabetes: with HbA1_C_ levels ≥6.5% (≥48 mmol/mol) being indicative of diabetes, and HbA1_C_ between 5.7-<6.5% (39 and <48mmol/mol) indicating a pre-diabetic state within which an individual is at a high risk of developing diabetes.[4]

There is heterogeneity within the results of studies which have sought to explore the associations between raised HbA1_C_ and all-cause mortality; with some finding an increased risk of mortality when compared with those who are normoglycaemic, while others demonstrate no difference[5–9]. The evidence is suggestive of a continuous association between HbA1_C_ and cardiovascular disease (CVD), including for myocardial infarction and stroke.[10,11] Khaw et al. found that the association between HbA1_C_ and mortality from all-causes and cardiovascular disease was not only continuous but occurred at measurements lower than the diabetic threshold.[12] A small number of published epidemiological studies have focussed upon HbA1_C_ and cancer mortality, with the majority demonstrating a positive association between the two,[13–15] although some did not.[16] Joshu et al. found that women with raised HbA1_C_ were at an increased risk of both cancer incidence and mortality, but this association was not present for men.[14]

There is also inconsistency in current research in a number of areas related to HbA1C and mortality, including whether there are differences in excess mortality related to HbA1_C_ between the sexes,[17–20] whether HbA1_C_ begins to impact upon mortality within the normoglycaemic range,[17,21,22] how raised HbA1_C_ impacts upon mortality among those with and without diagnosed diabetes—the latter being an indicator of how well an individual is controlling their diabetes,[22] and how it might impact upon mortality from site-specific cancers.[17,19,21] The impact that socioeconomic status has upon the excess mortality experienced by those with raised HbA1_C_/diagnosed diabetes has also yet to be fully assessed.[23]

We explored the associations between raised HbA1_C_ (within both the pre-diabetic and diabetic ranges) and mortality from all-causes, cancer and CVD. The impact of confounding factors, such as BMI and socioeconomic status, upon the associations were also assessed. Finally, in order to evaluate whether raised HbA1_C_ and diabetes impart mortality risks that differ between men and women, we performed sex-stratified analyses.

## Materials and Methods

### Participants and data

We linked data from 22,106 participants of the Health Survey for England (HSE) (survey years 2003, 2004, 2005, 2006 and 2008) with a valid HbA1_C_ measurement with UK national mortality data up to March 2013. Detailed descriptions of the HSE have been published previously.[24,25] Each year the HSE surveys a new, nationally-representative, random sample of the population utilising a multistage stratified design. Stage one of the HSE involves an interview within which information is sought about the participant’s health and lifestyle, and objective measurements of weight and height are taken. This is then followed by a visit from a nurse who collects information about medication use and takes further measurements and samples—including blood from which HbA1_C_ was measured. Analysis of the HbA1_C_ samples took place using the Tosoh G7 analyser calibrated to Diabetes Control and Complications Trial (DCCT) standards.[26] In total, 72% of men and 67% of women who took part in the nurse survey gave a valid non-fasting blood sample.[27]

Within the current study, an HbA1_C_ measurement of 6.5% (≥48mmol/mol) was considered indicative of the presence of diabetes, while a measurement of 5.7 to <6.5% (39 to <48mmol/mol) indicated pre-diabetes. These cut-off points are those recommended by the World Health Organization for the clinical diagnosis of diabetes, although in some instances a secondary measurement may also be required.[28] Diagnosed diabetes was ascertained using self-reported doctor diagnosed diabetes and/or recorded use of diabetic medicine during the nurse visit. We used this information, combined with the International Expert Committee’s diabetes and pre-diabetes categories, to create the following categorisation for those without diagnosed diabetes:

- Normoglycaemic/no diabetes: those who neither indicated diabetes nor had a raised HbA1_C_ measurement (<5.7% (<39mmol/mol))
- Pre-diabetes: those without diabetes but an HbA1_C_ measurement within the pre-diabetic range (5.7-<6.5% (39-<48mmol/mol))
- Undiagnosed diabetes: those with raised HbA1_C_ (≥6.5% (≥48mmol/mol)) without diagnosed diabetes.

The following categories were produced for individuals who indicated diagnosed diabetes:

- Controlled diabetes: those with diabetes and an HbA1_C_ <5.7% (<39mmol/mol).
- Diabetes and moderately raised HbA1_C_: those with diagnosed diabetes and an HbA1_C_ 5.7-<6.5% (39-<48mmol/mol).
- Uncontrolled diabetes: those with diagnosed diabetes and raised (≥6.5% (≥48mmol/mol)) HbA1_C_.

Smoking was categorised into non-regular-smokers, current and ex-smokers. The socioeconomic status (SES) variable used the Registrar-General’s Social Class categories based on occupation (I: Professional occupations, II: Managerial and technical, IIINM: Skilled non-manual, IIIM: Skilled manual, IV: Partly-skilled, V: Unskilled and other) were used. BMI (body mass index, based on measured height and weight) was included within the regression model as a continuous variable.

Variables were created for mortality from all-causes, as well as cancer (ICD9 codes 140–208; ICD10 codes C00-C97) and cardiovascular disease (390–459; I00-I99). Mortality data were categorised and linked to the HSE data by NatCen Social Research. Date of death was provided by quarter of a year and date of participation within the survey by month.

To be eligible for inclusion within this study, survey participants had to be 16 years or older and have given written consent for their participation within the HSE and for their data to be linked within national mortality records -80 to 95% of participants gave consent for this linkage. No children were included within the study, so consent was not required for their participation. Mortality records were available only for deaths that occurred within the UK. Ethical approval was obtained from the National Research Ethics Services.

### Statistical analysis

We used Cox regression to estimate models of the associations between glucose metabolism status and mortality. We adjusted our models for age, sex and smoking status. To assess the impact of socioeconomic status and overweight and obesity on the examined associations we progressively adjusted our models for socioeconomic status and BMI (as detailed above). Primary outcome measures were mortality from all-causes, cancer and CVD. All analyses were undertaken using SPSS V. 20 (SPSS Inc.). Follow-up time was defined as the period of time from the date of interview to either the end of the study period (March, 2013) or mortality as recorded within the data.

## Results

### Characteristic of the study sample

In total, 22,106 individuals aged ≥16 years within the HSE had a valid measurement for HbA1_C_. The average number of follow-up years was 7 (±SD 2.2). 54% of the sample were women. The sample was evenly split between never regular cigarette smokers and ex or current smokers. 15,476 (70) had neither a raised HbA1_C_ measurement nor diagnosed diabetes (see Table 1). Among those who reported diagnosed diabetes, 732 were in the uncontrolled diabetes category (HbA1_C_ ≥48mmol/mol (≥6.5)), 53 had controlled diabetes (HbA1_C_ <5.7% (<39mmol/mol)), and 234 had diabetes and moderately raised HbA1_C_ 5.7-<6.5% (39-<48mmol/mol). Of those without diagnosed diabetes, 15,476 had an HbA1_C_ within the normoglycaemic range 5.7% (<39mmol/mol). This category was used as our reference group. 413 had undiagnosed diabetes (HbA1_C_ ≥6.5% (≥48mmol/mol) but no diagnosis of diabetes) and 5,198 had pre-diabetes (HbA1_C_ 5.7-<6.5% (39mmol/mol-<48mmol/mol) and no diagnosis of diabetes). Those with raised HbA1_C_ tended to be older than those with a lower measurement; for example the mean age of those within the normoglycaemic range, and who did not report diabetes, was 47 years (±SD 0.14), while among those with uncontrolled diabetes the mean age was 64 (0.50). BMI was raised among those in either the diabetic or pre-diabetic groups when compared with those who were normoglycaemic.

**Table 1:** Characteristics of the study sample.

Total sample	22,106
Age: mean (SD)	52 (17.7)
Sex (%)	
Male	10,198 (46)
Female	11,908 (54)
Smoking status (%)	
Never regular	11,184 (51)
Current	4,489 (20)
Ex-regular	6,400 (29)
Missing	33 (<1)
HbA1_C_: mean (SD)	5.52 (0.74)
HbA1_C_: mean (SD) by diabetes category: n (%)	
Non-diabetic^1^	5.22 (0.002)15,476 (70)
Pre-diabetic^2^	5.89 (0.003)5,198 (24)
Undiagnosed diabetes^3^	7.46 (0.07)413 (2)
Controlled diabetes^4^	5.37 (0.03)53 (<1)
Diabetes and moderately raised HbA1_C_ ^5^	6.10 (0.01)234 (1)
Uncontrolled diabetes^6^	8.01 (0.05)732 (3)
BMI: mean (SD)	27 (4.8)
Missing	1160
Social Class (%)	
Social Class: I	1,233 (6)
Social Class: II	6,893 (31)
Social Class: IIINM	4,968 (23)
Social Class: IIIM	3,815 (17)
Social Class: IV	3,394 (15)
Social Class: V	1,054 (5)
Other	735 (3)
Missing	14 (<1)
Mortality (%)	
Alive	20,597 (93)
Dead	1,509 (7)
Cause of death (%)	
Cancer	466 (2)
CVD	506 (2)

^1^ Normoglycaemic/no diabetes: those who neither indicated diabetes nor had a raised HbA1_C_ measurement (<39mmol/mol (<5.7%))

^2^ Pre-diabetes: those without diabetes but an HbA1_C_ measurement within the pre-diabetic range (39-<48mmol/mol (5.7-<6.5%)).

^3^ Undiagnosed diabetes: those with raised HbA1_C_ (≥48mmol/mol (≥6.5%)) without diagnosed diabetes

^4^ Controlled diabetes: those with diabetes and an HbA1_C_ <39mmol/mol (5.7%).

^5^ Diabetes and moderately raised HbA1_C_: those with diagnosed diabetes and an HbA1_C_ 39-<48mmol/mol (5.7–6.49%).

^6^ Uncontrolled diabetes: those with diagnosed diabetes and raised (≥48mmol/mol (≥6.5%)) HbA1_C_.

1,509 participants (6.8% of the sample) had died by the end of the follow-up period; 506 participants had died of CVD and 466 from cancer.

### All-cause mortality

**Fig 1:**
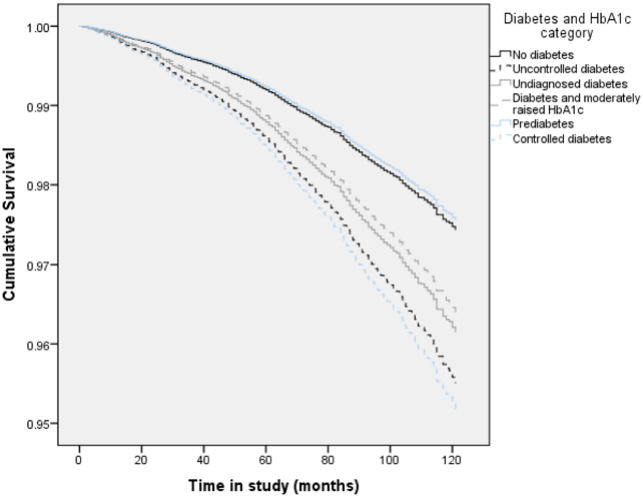
Fully adjusted (age, sex, smoking, SES and BMI) all-cause mortality survival curves by HbA1_C_ and diabetes category (whole sample).

Compared with those without diabetes, participants with undiagnosed, uncontrolled, or diabetes with moderately raised HbA1_C_ had an increased HR in relation to all-cause mortality; although those with pre-diabetes or controlled diabetes did not (see Table 2). When the analyses were stratified by sex, women who had either undiagnosed or uncontrolled diabetes were at an increased risk of all-cause mortality 1.61 (1.12–2.30) and 2.27 (1.72–3.01), respectively) while only men with uncontrolled diabetes were found to have an increased HR (1.62, 1.27–2.05) (Table 3). Results were not significantly altered by adjustment for SES and BMI. Fig. 1 demonstrates the fully adjusted (age, sex, smoking status, SES and BMI) survival curves for cancer mortality HbA1_C_ and diabetes category.

**Table 2:** HRs for all-cause and cause-specific mortality by glycated haemoglobin and self-reported diabetes category.

	n	All-cause	Cancer	CVD
**Age, sex & smoking**	22,073			
No diabetes^1^		1	1	1
Pre-diabetes^2^		0.93(0.83–1.04)	0.88(0.72–1.08)	1.07 (0.88–1.32)
Undiagnosed diabetes^3^		1.40(1.09–1.80)	1.46(0.94–2.27)	1.99(1.35–2.94)
Controlled diabetes^4^		1.71(0.89–1.04)	0.61(0.09–4.38)	3.22(1.32–7.84)
Diabetes and moderately raised HbA1_C_ ^5^		1.42(1.07–1.90)	1.03(0.56–1.89)	1.89(1.20–2.97)
Uncontrolled diabetes^6^		1.85(1.54–2.22)	1.24(0.85–1.81)	2.63(1.97–3.51)
**+ SES**	22,060			
No diabetes^1^		1	1	1
Pre-diabetes^2^		0.92(0.82–1.03)	0.87(0.70–1.07)	1.07(0.87–1.31)
Undiagnosed diabetes^3^		1.38(1.07–1.78)	1.430.92–2.22)	1.98(1.34–2.93)
Controlled diabetes^4^		1.69(0.88–3.27)	0.61(0.09–4.38)	3.16(1.30–7.69)
Diabetes and moderately raised HbA1_C_ ^5^		1.39(1.05–1.85)	1.01(0.55–1.85)	1.84(1.17–2.90)
Uncontrolled diabetes^6^		1.80(1.50–2.16)	1.20(0.82–1.75)	2.57(1.93–3.44)
**+ BMI**	20,906			
No diabetes^1^		1	1	1
Pre-diabetes^5^		0.95(0.84–1.08)	0.88(0.71–1.10)	1.06(0.85–1.31)
Undiagnosed diabetes^3^		1.51(1.15–1.97)	1.40(0.87–2.26)	2.07(1.37–3.15)
Controlled diabetes^6^		1.90(0.98–3.68)	0.67(0.09–4.76)	3.43(1.41–8.37)
Diabetes and moderately raised HbA1_C_ ^4^		1.41(1.02–1.94)	0.86(0.42–1.74)	1.81(1.10–3.00)
Uncontrolled diabetes^2^		1.77(1.44–2.16)	1.19(0.79–1.79)	2.32(1.68–3.22)

^1^ Normoglycaemic/no diabetes: those who neither indicated diabetes nor had a raised HbA1_C_ measurement (<39mmol/mol (<5.7%))

^2^ Pre-diabetes: those without diabetes but an HbA1_C_ measurement within the pre-diabetic range (39-<48mmol/mol (5.7-<6.5%)).

^3^ Undiagnosed diabetes: those with raised HbA1_C_ (≥48mmol/mol (≥6.5%)) without diagnosed diabetes

^4^ Controlled diabetes: those with diabetes and an HbA1_C_ <39mmol/mol (5.7%).

^5^ Diabetes and moderately raised HbA1_C_: those with diagnosed diabetes and an HbA1_C_ 39-<48mmol/mol (5.7–6.49%).

^6^ Uncontrolled diabetes: those with diagnosed diabetes and raised (≥48mmol/mol (≥6.5%)) HbA1_C_.

**Table 3:** HRs for all-cause and cause-specific mortality by glycated haemoglobin and diagnosed diabetes category, stratified by sex.

	n^1^	All-cause		Cancer		CVD	
**Age, sex & smoking**	11,89210,181	Women	Men	Women	Men	Women	Men
No diabetes		1	1	1	1	1	1
Pre-diabetes		0.97(0.82–1.14)	0.90(0.77–1.06)	1.13(0.83–1.54)	0.71(0.53–0.94)	1.09(0.81–1.47)	1.08(0.82–1.43)
Undiagnosed diabetes		1.61(1.12–2.30)	1.25(0.88–1.78)	1.13(0.49–2.59)	1.64(0.98–2.76)	2.78(1.65–4.69)	1.41(0.78–2.57)
Controlled diabetes		1.61(0.66–3.91)	1.76(0.66–4.72)	1.47(0.20–10.61)	N/A	1.90(0.46–7.77)	4.55(1.44–14.32)
Diabetes and moderately raised HbA1_C_		1.38(0.90–2.14)	1.45(0.99–2.12)	1.37(0.55–3.39)	0.85(0.37–1.92)	1.39(0.64–3.01)	2.28(1.30–3.98)
Uncontrolled diabetes		2.27(1.72–3.01)	1.62(1.27–2.05)	1.57(0.84–2.94)	1.08(0.68–1.73)	3.51(2.26–5.45)	2.24 (1.53–3.28)
**+SES**	11,88110,166						
No diabetes		1	1	1	1	1	1
Pre-diabetes		0.95(0.80–1.11)	0.89(0.75–1.04)	1.10(0.81–1.50)	0.69(0.52–0.92)	1.08(0.80–1.46)	1.08(0.81–1.42)
Undiagnosed diabetes		1.54(1.07–2.20)	1.26(0.88–1.79)	1.06(0.46–2.43)	1.69(1.01–2.84)	2.71(1.60–4.58)	1.42(0.78–2.58)
Controlled diabetes		1.60(0.66–3.90)	1.67(0.62–4.48)	1.49(0.21–10.76)	N/A	1.80(0.44–7.38)	4.41(1.40–13.90)
Diabetes and moderately raised HbA1_C_		1.32(0.85–2.03)	1.46(1.00–2.13)	1.28(0.52–3.16)	0.87(0.38–1.97)	1.34(0.62–2.92)	2.25(1.29–3.94)
Uncontrolled diabetes		2.19(1.66–2.90)	1.57(1.23–1.99)	1.48(0.79–2.77)	1.04(0.65–1.67)	3.42(2.22–5.32)	2.19(1.50–3.21)
**+ BMI**	11,1969,698						
No diabetes		1	1	1	1	1	1
Pre-diabetes		0.99(0.83–1.18)	0.92(0.77–1.09)	1.11(0.80–1.53)	0.71(0.53–0.97)	1.06(0.76–1.46)	1.08(0.81–1.45)
Undiagnosed diabetes		1.71(1.16–2.51)	1.35(0.93–1.97)	1.16(0.50–2.70)	1.60(0.90–2.84)	2.67(1.51–4.72)	1.58(0.85–2.97)
Controlled diabetes		1.87(0.76–4.56)	1.87(0.70–5.01)	1.66(0.23–11.98)	N/A	2.04(0.49–8.45)	4.90(1.55–15.50)
Diabetes and moderately raised HbA1_C_		1.46(0.90–2.37)	1.37(0.90–2.10)	1.53(0.61–3.81)	0.50(0.16–1.57)	1.23(0.49–3.06)	2.27(1.24–4.16)
Uncontrolled diabetes		2.25(1.65–3.09)	1.52(1.16–1.98)	1.38(0.69–2.78)	1.06(0.64–1.75)	3.40(2.08–5.52)	1.90(1.23–2.95)

^1^The first number relates to the total number of women included in the analyses, while the second relates to the number of men.

### Cancer and cardiovascular disease mortality

**Fig 2:**
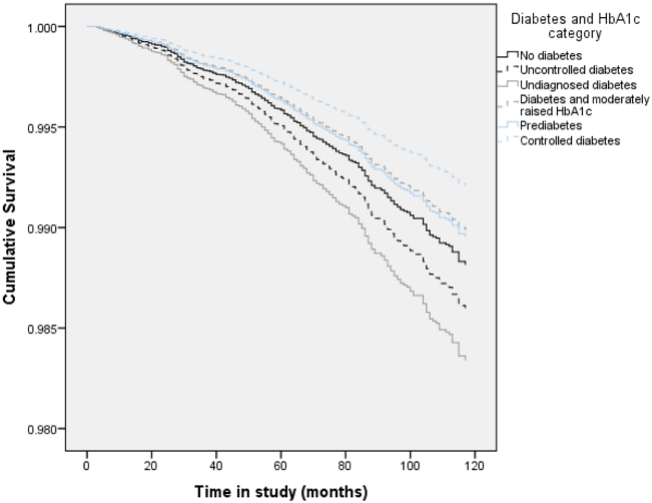
Fully adjusted survival curves for cancer mortality by HbA1_C_ and diabetes category (whole sample).

Raised HbA1_C_ and/or diabetes did not confer an increased risk of cancer mortality when compared with those without diabetes; this result was also found within the sex-stratified analyses (Table 2). Fig. 2 details the survival curves for cancer mortality by HbA1_C_/diabetes category.

**Fig 3:**
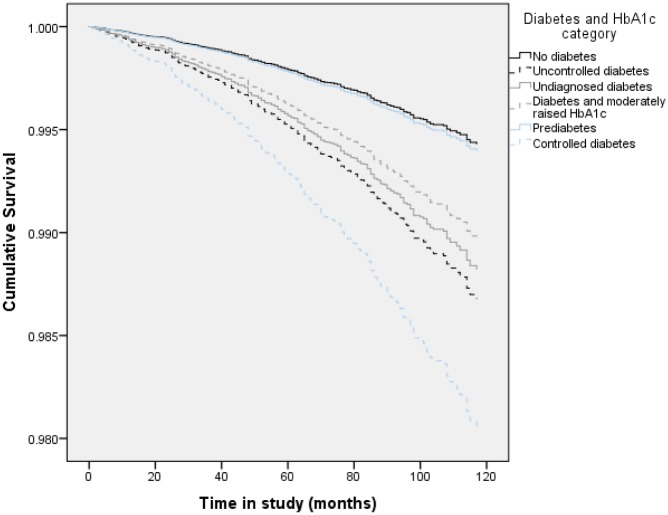
Fully adjusted survival curves for CVD mortality by HbA1_C_ and diabetes category (whole sample).

The situation was different for cardiovascular mortality (Table 2). Compared with those without diabetes, each group categorised as having abnormal HbA1_C_ or diabetes had a statistically significant excess in mortality from CVD apart from those with pre-diabetes. For each group (undiagnosed, controlled diabetes, uncontrolled, and those with diabetes and moderately raised HbA1_C_) the HRs were substantially increased (1.99 (1.35–2.94), 3.22 (1.32–7.84), 2.63 (1.97–3.51), and 1.89 (1.20–2.97), respectively). The elevation in risk survived adjustment for SES and BMI. Fig. 3 details the survival curves for CVD mortality by HbA1_C_/diabetes category. When the analyses were sex-stratified, we observed a mixed pattern. Women with undiagnosed or uncontrolled diabetes had an increased risk of mortality from CVD, while for men, controlled diabetes, diabetes with moderately raised HbA1_C_ and uncontrolled diabetes were associated with excess CVD mortality. The elevated mortality risk for men with controlled diabetes was particularly striking. None of these results were materially altered by adjustment for SES or BMI.

## Discussion

Our study demonstrated an excess in mortality from all-causes among those with raised HbA1_C_, including within the pre-diabetes range among those with diagnosed diabetes. Raised HbA1_C_ among people with diabetes was also strongly associated with mortality from CVD, but not cancer. BMI and socioeconomic status did not affect the associations.

These results support those of Skriver et al. who found increased all-cause mortality among those with an HbA1_C_ measurement ≥7% (53 mmol/mol) (HR 1.26 (95% CI 1.15, 1.39)) and a U-shaped relationship between increasing HbA1_C_ and all-cause mortality.[8] A 2013 study also found an increased HR in relation to HbA1_C_ and all-cause mortality that was marginally non-significant (HR 1.21, 95% CI 0.99–1.47).[5] Within the current study, among those with diabetes only those with raised HbA1_C_ had an increased risk of all-cause mortality, while for mortality from CVD all diabetes/raised HbA1_C_ categorieshad an increased risk. The only exception to this was pre-diabetes, which was not associated with any of the causes of mortality under investigation; this result contradicts those of a recent meta-analysis, which found associations between pre-diabetes and mortality from all-causes and CVD, suggesting the need for further analyses in this area.[29]

The results of our current study support those of previous research in finding HbA1_C_ to be more strongly associated with mortality from all-causes and CVD rather than cancer.[5,10,15] Compared with normoglycaemic participants, those with raised HbA1_C_ and/or diabetes (uncontrolled, undiagnosed or with a moderately raised HbA1_C_) had an increased risk of mortality from all-causes and CVD; those with controlled diabetes had an increased risk of CVD mortality only. Earlier studies found that impaired glucose metabolism impacts upon CVD mortality for women to a greater extent than for men[30]; our results indicate differences in risk by sex dependent upon HbA1_C_/diabetes category analysed.

We found HbA1_C_ and diabetes were not significantly associated with cancer mortality, although an earlier cohort study found a 1mmol/l increment in glucose increased the risk of fatal cancer for both men (1.15, 1.07–1.22) and women (1.21, 1.11–1.33).[18] Joshu et al. also found that raised HbA1_C_ was not associated with increased cancer mortality among men, but was among women.[31] Research able to utilise larger datasets with a greater number of cancer mortality endpoints is needed to further assess the association between HbA1_C_ and cancer mortality. One reason for the lack of association between HbA1_C_ and cancer mortality within our results, but found in those of earlier studies, may be the differing rates of site-specific cancers within the all-cancers outcome variable used within each study. Although limited in number, previous studies have suggested that increased blood glucose is associated with increased mortality from a number of site-specific cancers, but is not associated with others.[32] Therefore results related to a grouped, all-cancers variable may be concealing differences in site-specific cancer mortality risk. Further research is required which explores the associations between HbA1_C_ and site-specific cancer mortality and incidence. The larger sample size utilised by Stocks et al.,[18] and the longer follow-up time and corresponding greater number of cancer deaths in the research undertaken by Joshu et al.,[31] compared with our study may also have given these studies the power needed to find a small, statistically significant difference. Although a power calculation undertaken before commencement of this current study suggested that our analyses were not underpowered, future studies which are able to utilise larger samples and investigate the sex differences within the associations between impaired glucose metabolism and mortality are required.

A number of plausible pathways have been proposed for the relationship between HbA1_C_, diabetes and cause-specific mortality. Increased rates of atherosclerosis appear to explain much of the association between diabetes and cardiovascular disease; raised HbA1_C_ has been found to be strongly associated with carotid intima-media thickness, a marker of atherosclerosis.[33,34]. Although the epidemiological evidence related to HbA1_C_ and cancer is limited, the key biological causal factors relate to the anti-apoptotic, proliferative and mitogenic nature of insulin and insulin-like growth factor (IGF) and the impact that hyperglycaemia has upon mortality risk.[35–37] If a causal association were to be found between raised HbA1_C_ and mortality from CVD and other causes then some of the differences found in mortality between those with and without diabetes could be overcome through stricter glycaemic control. At the same time the potential deleterious impact of tighter control—for example the increased mortality found within the strict glucose control group of the Action to Control Cardiovascular Risk in Diabetes (ACCORD) study and the risk of hypoglycaemia [9,38–40]—would have to be balanced against any such reductions in mortality and take into account the specific characteristics of each individual with raised blood glucose.[9,41]

A strength of this study was its utilisation of a nationally-representative, general population sample of over 22,000 participants with a recorded HbA1_C_ measurement and comprehensive linked mortality data. The use of HSE data enabled the inclusion of a detailed HbA1_C_ and self-reported diabetes variable and a range of confounding factors within the analyses. Further, unlike a number of studies which have utilised only self-reported diabetes as an indicator of the presence of the disease, this study used a valid measure of HbA1_C_ to explore the association between diabetes, blood glucose and mortality. The recording of the use of diabetes-related medications, within the nurse visit, also enabled the further verification of diagnosed diabetes information.

The majority of the study sample self-identified as white—around 85%—a similar percentage to the general, free-living population of England.[42] Although the results of earlier analyses (not shown) were suggestive of ethnicity not having a significant impact upon the excess mortality found among those with raised HbA1_C_/diabetes, the small number of deaths among those from Black and Minority Ethnic populations and the corresponding lack of power made interpretation of the results difficult. Studies utilising larger datasets, with a corresponding larger number of mortality endpoints, could further explore the impact of ethnicity.

The current study did not assess the impact that the use of different types of diabetes-related medications may have upon the excess risk experienced by those with diabetes. Those with diabetes may change drug regimens regularly and may switch from oral medications to insulin as their disease progresses. Because of this, research in this area favours the use of randomised controlled trials to explore this issue further.

Our results demonstrate that diabetes/raised HbA1_C_ are associated with mortality from CVD and all-causes. This result was found among those with and without diagnosed diabetes and, if confirmed, suggests that the monitoring of impaired blood glucose could be an important tool in relation to reducing mortality from these causes. The results also support the introduction of clinical practices which aim to prevent and detect pre-diabetes, diagnose previously undiagnosed diabetes, and optimise HbA1_C_ levels among people with diagnosed diabetes.

## Conclusion

This study—utilising data from a nationally representative sample—demonstrates an association between HbA1_C_ and all-cause and cardiovascular disease mortality among those with and without diagnosed diabetes that remains after adjustment for a range of confounding factors including adiposity. If these result were to be replicated within further studies, the lowering of HbA1_C_ within populations could become an important public health goal.
